# Graphene Oxide-Gold Nanorods Nanocomposite-Porphyrin Conjugate as Promising Tool for Cancer Phototherapy Performance

**DOI:** 10.3390/ph14121295

**Published:** 2021-12-11

**Authors:** Thabang Calvin Lebepe, Sundararajan Parani, Vuyelwa Ncapayi, Rodney Maluleke, Grace It Mwad Mbaz, Olufunto Tolulope Fanoro, Jose Rajendran Varghese, Atsuki Komiya, Tetsuya Kodama, Oluwatobi Samuel Oluwafemi

**Affiliations:** 1 Department of Chemical Science, University of Johannesburg, Johannesburg 2028, South Africa; calvyn.tl@gmail.com (T.C.L.); sbarani416@gmail.com (S.P.); vuyelwa.tito.ncapayi530@gmail.com (V.N.); rodney.maluleke@gmail.com (R.M.); gmbazitmwad@gmail.com (G.I.M.M.); josv3209@gmail.com (J.R.V.); 2 Centre for Nanomaterials Sciences Research, University of Johannesburg, Johannesburg 2028, South Africa; jolufunto@gmail.com; 3 Institute of Fluid Science, Tohoku University, Sendai 980-8577, Japan; komiya@tohoku.ac.jp; 4 Graduate School of Biomedical Engineering, Tohoku University, Sendai 980-8575, Japan; kodama@tohoku.ac.jp

**Keywords:** graphene oxide, gold nanorods, TMePyP, photothermal, singlet oxygen, cytotoxicity

## Abstract

The cancer mortality rate has increased, and conventional cancer treatments are known for having many side effects. Therefore, it is imperative to find a new therapeutic agent or modify the existing therapeutic agents for better performance and efficiency. Herein, a synergetic phototherapeutic agent based on a combination of photothermal and photodynamic therapy is proposed. The phototherapeutic agent consists of water-soluble cationic porphyrin (5,10,15,20-tetrakis(*N*-methylpyridinium-3-yl)porphyrin, TMePyP), and gold nanorods (AuNRs) anchored on graphene-oxide (GO) sheet. The TMePyP was initially synthesized by Adler method, followed by methylation, while GO and AuNRs were synthesized using Hummer’s and seed-mediated methods, respectively. The structural and optical properties of TMePyP were confirmed using UV-Vis, zeta analyzer, PL, FTIR and NMR. The formation of both GO and AuNRs was confirmed by UV-Vis-NIR, FTIR, TEM and zeta analyzer. TMePyP and AuNRs were anchored on GO to form GO@AuNRs-TMePyP nanocomposite. The as-synthesized nanocomposite was stable in RPMI and PBS medium, and, on irradiation, produced high heat than the bare AuNRs, with high photothermal efficiency. In addition, the nanocomposite produced higher singlet oxygen than TMePyP with high biocompatibility in the absence of light. These results indicated that the as-synthesized nanocomposite is a promising dual photodynamic and photothermal agent for cancer therapy.

## 1. Introduction

Cancer synergistic therapy is gaining the spotlight as statistics of cancer incidents are anticipated to increase by 38.6% by 2040, according to the World Health Organization [1]. Synergistic cancer therapy has an advantage as it is the combination of multiple therapies to avoid cancer reoccurrence [2]. However, some of these synergistic therapies, such as combined chemotherapy/immunotherapy, can cause side effects, leading to patients scarring and drug resistance. Phototherapeutic treatments such as photothermal and photodynamic have been shown to be non-invasive, highly comprehensive, and precise with low side effects [3,4,5]. In phototherapy, high penetration into biological tissues is a requirement. Nanomaterials must absorb in the near-infrared (NIR) region, with wavelengths ranging from 650–1350 nm [3,6], and gold nanorods (AuNRs) are of excellent choice.

AuNRs can be easily tuned to absorb in different NIR ranges (650–1100 nm) [7]; however, most AuNRs are prone to induce toxicity when used in biological applications. Researchers have used different coating materials, such as polymers [8,9,10,11,12,13,14,15,16], biomolecules [17,18,19] and graphene-based materials [15,20,21,22,23], to reduce the toxicity of AuNRs. Among them, graphene-based AuNRs are more effective as they reduce toxicity and simultaneously increase their photothermal efficiency [15,20,21,22,23]. AuNRs have also been investigated for photo synergistic therapy using different photosensitisers such as chlorin e6 [5,24,25], phthalocyanine [26], rose Bengal [27], indocyanine green [28], 5,10,15,20-tetrakis(N-methylpyridinium-4-yl)porphyrin tosylate salt [29], pheophorbide *a* [30] and sulfonated aluminium phthalocyanines [31]. The combination of AuNRs and photosensitizers could yield a synergistic photothermal and photodynamic system.

**Scheme 1:**
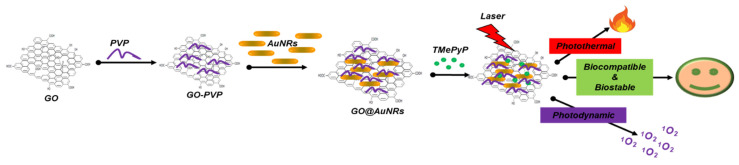
Schematic illustration of the preparation and properties of the GO@AuNRs-Porphyrin conjugate.

Since the clinical approval of hematoporphyrin derivative (HpD) as a photosensitizer for photodynamic therapy, porphyrin derivatives have been synthesized and investigated for photodynamic therapy application. Among this porphyrin, 5,10,15,20-tetrakis (3-methyl pyridyl) porphyrin (TMePyP) has been reported to be an efficient photosensitizer due to its strong ability to produce reactive oxygen species, which could bind to the DNA G-quadruplex in the cancer cell and temper the cell’s cycle, leading to apoptosis or senescence [32,33]. Nevertheless, for TMePyP to produce effective reactive oxygen species, it requires high oxygen concentration, which restricts its therapeutic effect [32]. In addressing this issue, we reported the conjugation of modified GO coated AuNRs to this photosensitizer to produce a photo synergistic therapeutic agent. As far as the authors know, such a synergistic therapeutic agent has not yet been reported. In this study, TMePyP anchored on GO-coated AuNRs nanocomposite was fabricated to achieve synergistically enhanced cancer phototherapy. Firstly, a water-soluble porphyrin (TMePyP) was synthesized, and on the other hand, AuNRs synthesized via seed-mediated method were incorporated on polyvinylpyrrolidone (PVP) modified GO (GO@AuNRs). The as-synthesized water-soluble porphyrin was anchored on the GO@AuNRs to form GO@AuNRs-TMePyP nanocomposites. The AuNRs incorporated on the modified GO exhibited good photothermal efficiency and stability. The as-synthesized nanocomposite was found to be stable in different media, with an improved singlet oxygen quantum yield generation compared to the bare TMePyP. The toxicity of all the as-synthesized materials was evaluated using MTT assay against mouse bladder cancer cell line (MBT-2), which showed the excellent biocompatibility of GO@AuNRs-TMePyP as a good photo synergistic therapeutic agent for biological applications (Scheme 1).

## 2. Results and Discussion

### 2.1. Characterization of 5,10,15,20-Tetrakis(1-methylpyridinium-3-yl)porphyrin

**Figure 1:**
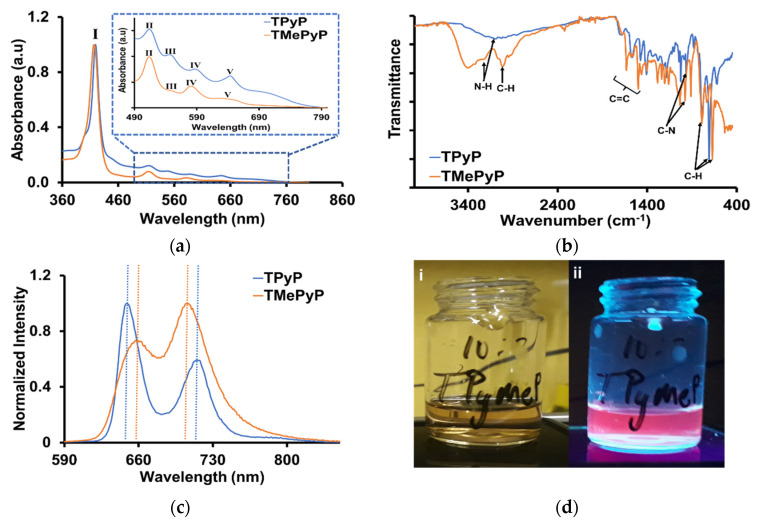
(**a**) UV-Vis spectra of TPyP and TMePyP with enlarged UV-Vis spectra. (**b**) FTIR spectra of TPyP and TMePyP. (**c**) Normalized PL spectra of TPyP and TMePyP excitation of 415 nm. (**d**) Images of TMePyP under normal light (**i**) and UV-light irradiation (**ii**).

The synthesis of 5,10,15,20-tetrakis(pyridinium-3-yl)porphyrin (TPyP) was carried out using the modified Adler method, and the product was methylated using methyl iodide to produce TMePyP as shown in Scheme S1. The formation of TPyP and its methylated derivative (TMePyP) was confirmed by UV-Vis, FTIR, NMR and PL analyses. UV-Vis spectrum of TPyP showed five characteristic peaks of porphyrin: the soret peak (I) at 420 nm, with four Q-bands at 516 nm (II), 550 nm (III), 588 nm (IV), and 646 nm (V), (Figure 1a) [34,35]. After methylation, the Soret peak-I was blue-shifted by 2 nm, whereas the Q-band (II) absorbance peak became sharp, which reduced the Q-band (III) to look like a shoulder. The Q-band (IV) was blue-shifted by 10 nm, and lastly, the Q-band (V) became broad, as seen in Figure 1a insert. These changes signal the methylation of TPyP. The as-synthesized TMePyP was readily soluble in water.

The FTIR (Figure 1b) of TPyP showed stretching vibrations of an aromatic amine group (N-H) and the bending of aromatic amine (C-N) at 3113 and 964 cm^−1,^ respectively, which are the characteristic absorptions of free-base porphyrin. After methylation of TPyP to form TMePyP, both N-H and C-N bands shifted to the higher wavenumber by 118 and 14 cm^−1,^ respectively. The asymmetric skeleton vibrations of the methyl bridges, pyrrole rings and the stretching vibration of C=C in the pyridyl aromatic ring are seen at bands around 1500–1600 cm^−1^. An intense band shows the vibration of the C-H bond from pyrrole at 793 cm^−1,^ which is seen in both porphyrins with no shift. A new C-H stretching mode of the methyl group at 3026 cm^−1^ is visible in the spectra of TMePyP, which confirmed the methylation of TPyP.

The ^1^H-NMR spectrum of TPyP in CDCl_3_ at 500 MHz shows a signal at −2.845 ppm corresponding to NH pyrrolic protons (Figure S1). The signals for H-Phenyl (meso) protons are four singlets at 6.77–7.768 ppm, whereas the signals for ortho 3,5 phenyl protons are the doublets between 8.178 and 8.531 ppm. The signals for β pyrrolic protons are located at 8.810–8.819 ppm. The two doublets signal peaks at 9.052–9.139 ppm are attributed to para phenyl protons. The ortho 2,6 phenyl protons singlet is located at 9.449 ppm. The signal peaks at 8.810 ppm and 0.068 ppm are due to the solvent used, CDCl_3_ and water, respectively. The ^1^H-NMR spectrum of TPyP presents all the expected signals. The asymmetrical meso substituted compound in which a significant diminution of the current of the cycle causes the shielding of β pyrrolic protons and the un-shielding of internal NH protons [36]. The ^1^H-NMR spectrum of TMePyP (Figure S2) shows new peaks at 1.750–3.119 ppm compared to TPyP due to methylation. The signals in the ^13^C-NMR of TPyP are demonstrated in Figure S3 and are divided into some major regions. The α-pyrrolic carbons, which are very sensitive to electronic effects, display a signal between 149 and 153.57 ppm. The β-pyrrolic carbons resonate around 137.67–140.95 ppm and the meso-carbons between 116.74 ppm and 123.63 ppm. The three signals at 76.78–77.29 ppm are from the solvent CDCl_3_. Figure S4 shows the ^13^C-NMR signals of TMePyP, which are similar to TPyP. The porphyrins were further analyzed using PL. The emissions were seen at 650 nm and 716 nm for TPyP, and 661 nm and 708 nm for TMePyP, respectively (Figure 1c). The TMePyP was soluble in water (Figure 1d), showing brown colour under the daylight and emitted a red-pink colour under UV-light (λ = 365 nm).

### 2.2. Synthesis of GO and Its Surface Modification with PVP

**Figure 2:**
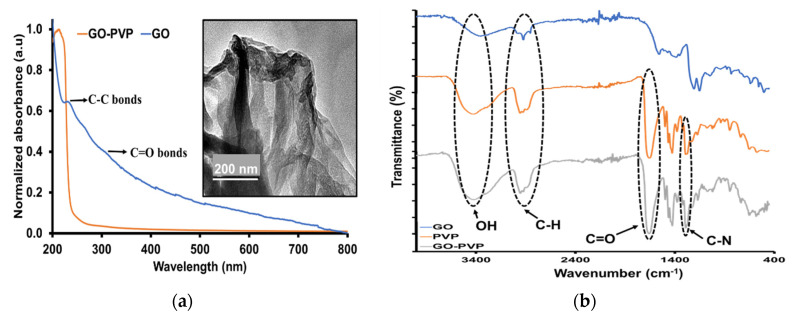
(**a**) UV-Vis spectra of GO and GO-PVP (Inset GO-PVP TEM image scale 200 nm), and (**b**) FTIR spectra of GO, PVP and GO-PVP.

GO was prepared using the Hummers method and then modified with PVP to increase the aqueous dispersibility [37,38]. The UV spectrum of the as-synthesized GO (Figure 2a) showed small shoulder peaks corresponding to π-π* carbon-carbon and *n*-π* oxygen-carbon transitions [39]. When GO was modified with PVP, the π-π* and *n*-π* of GO disappeared, indicating the interaction of PVP with GO. To further prove this, FTIR spectra of GO, PVP and GO-PVP were analyzed, which demonstrated that PVP chains have completely masked GO sheet surface. This could be attributed to the fact that PVP and GO-PVP vibrations are similar, with both spectra showing two stretched peaks at 1670 and 1283 cm^−1^ corresponding to C=O and C–N of PVP, respectively (Figure 2b). The TEM image of GO-PVP inserted in Figure 2a portrayed a 2-dimensional sheet of GO.

### 2.3. Synthesis of AuNRs and Fabrication of GO@AuNRs Nanocomposite

**Figure 3:**
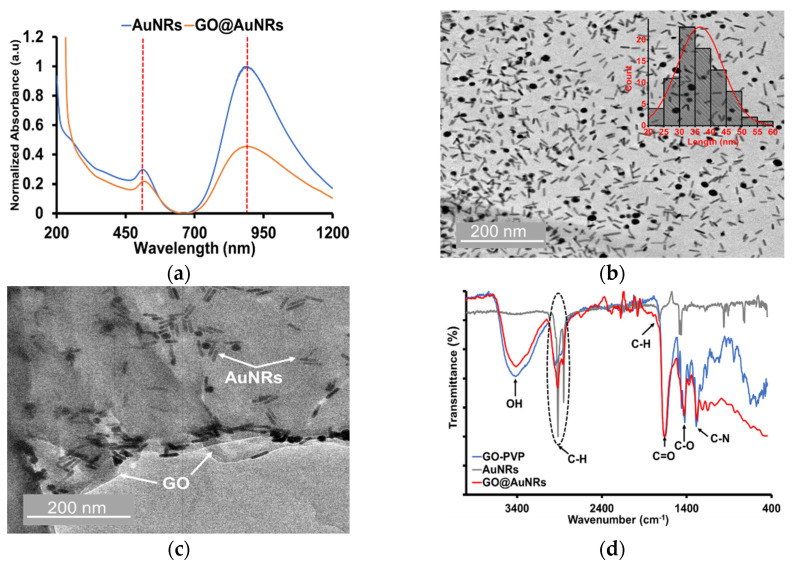
(**a**) UV-Vis spectra of AuNRs and GO@AuNRs. TEM image of (**b**) AuNRs with corresponding length distribution histogram and (**c**) GO@AuNRs (scale: 200 nm). (**d**) FTIR of GO-PVP, AuNRs and GO@AuNRs.

The AuNRs was synthesized using the seed-mediated method. The as-synthesized AuNRs was confirmed using UV-Vis-NIR spectroscopy and TEM. The UV-Vis-NIR spectrum showed two unique peaks at 512 nm and 850 nm, which were due to the transverse surface plasmon resonance (TSPR) and longitudinal surface plasmon resonance (LSPR), respectively (Figure 3a). The UV-Vis-NIR spectrum of GO@AuNRs composites shows the presence of AuNRs peaks. This indicates that the incorporation of AuNRs on the GO sheet did not affect their shape. The TEM image shows AuNRs with an average length of 36.60 nm and width of 5.96 nm, and few spherical particles with an average diameter of 15 nm (Figure 3b). TEM image of GO@AuNRs (Figure 3c) portrayed AuNRs widely anchored on the surface of GO. Figure 3d illustrated the FTIR spectra of PVP modified GO, AuNRs and GO@AuNRs. The characteristic peaks at ~3424 cm^−1^, 2860–2925 cm^−1^, ~1667 cm ^−1^, ~1240 cm ^−1^ and 1281 cm ^−1,^ which belong to O–H, C–H, C=O, C–O and C-N bond vibrations respectively in the PVP modified GO, can also be seen in the GO@AuNRs. Furthermore, the GO@AuNRs spectrum also shows the presence of alkyl C-H stretching vibration bands at ~2850 and ~2918 cm^−1^ [40], corresponding to the CTAB capping agent on the AuNRs.

### 2.4. Anchoring of Porphyrin on the GO@AuNRs Nanocomposite

**Figure 4:**
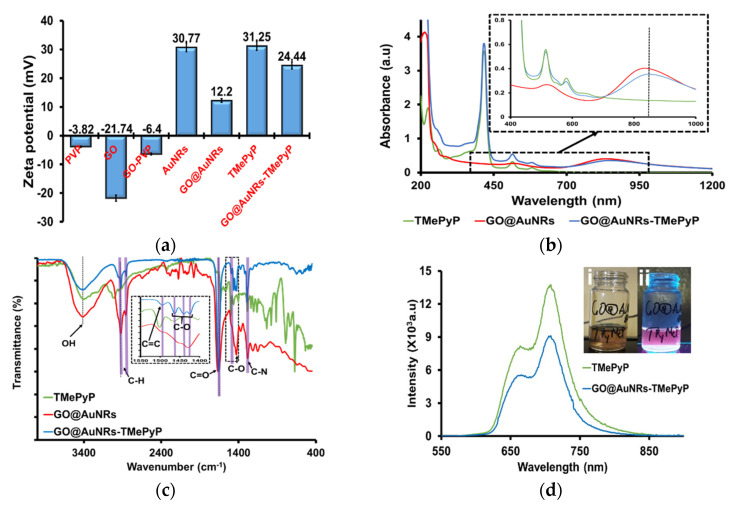
(**a**) Zeta potential of GO, PVP, GO-PVP, AuNRs, GO@AuNRs, TMePyP and GO@AuNRs-TMePyP. (**b**) UV-Vis-NIR spectra of Por, GO@AuNRs and GO@AuNRs-TMePyP, and inset of enlarged UV-Vis-NIR spectrum. (**c**) FTIR spectra of 3-TMePyP, GO@AuNRs and GO@AuNRs-TMePyP. (**d**) PL spectra of TMePyP and GO@AuNRs-TMePyP at an excitation wavelength of 415 nm, inset: images of GO@AuNRs-TMePyP under normal light (**i**) and UV-light irradiation (**ii**).

The zeta potential of PVP, GO, modified GO demonstrated a potential change from −22.74 for GO to −6.40 mV after modification with PVP (Figure 4a). Moreover, after incorporating positively charged AuNRs (+30.77 mV) on the partially negative charge modified GO, the potential changed to +12.20 mV due to the interaction between modified GO and AuNRs. When a water-soluble porphyrin (TMePyP) was anchored on the GO@AuNRs nanocomposite, the potential increased to +24.44 mV due to the cationic charge of TMePyP. The UV-Vis-NIR spectrum of GO@AuNRs-TMePyP in Figure 4b shows both peaks of AuNRs and porphyrin. However, the LSPR peak of AuNRs in GO@AuNRs-TMePyP was red-shifted by 20 nm. The TSPR of AuNRs merged with the Q-bands of porphyrin. Figure 4c shows the FTIR spectrum of GO@AuNRs-TMePyP, which exhibits most of the GO@AuNRs bands such as OH broadband, C=O and C-O stretching vibration at ~3424 cm^−1^, ~1667 cm^−1^ and ~1240 cm^−1,^ respectively. The alkyl C-H bands of AuNRs at ~2850 and ~2918 cm^−1^ became sharper in the nanocomposite due to the porphyrin alkyl C-H bands. The porphyrin peaks at 1494 cm^−1^ and 1291 cm^−1^ due to their C=C and C-N stretching can also be seen in the GO@AuNRs-TMePyP spectrum. These results indicate the formation of GO@AuNRs-TMePyP nanocomposite.

The photoluminescence (PL) spectrum (Figure 4d) of GO@AuNRs-TMePyP shows similar emission peaks of TMePyP at 661 and 708 nm, further confirming its presence in the nanocomposite. The decrease in intensity observed can be attributed to the intramolecular donor-acceptor interaction of TMePyP and graphene oxide through photoinduced electron transfer or energy transfer, which result in slight quenching [41,42]. When the GO@AuNRs-TMePyP solution was irradiated under UV-light, the red-pink colour emission of TMePyP was maintained (Figure 4d inset).

### 2.5. In vitro Photothermal Effect and Photostability of GO@AuNRs

**Figure 5:**
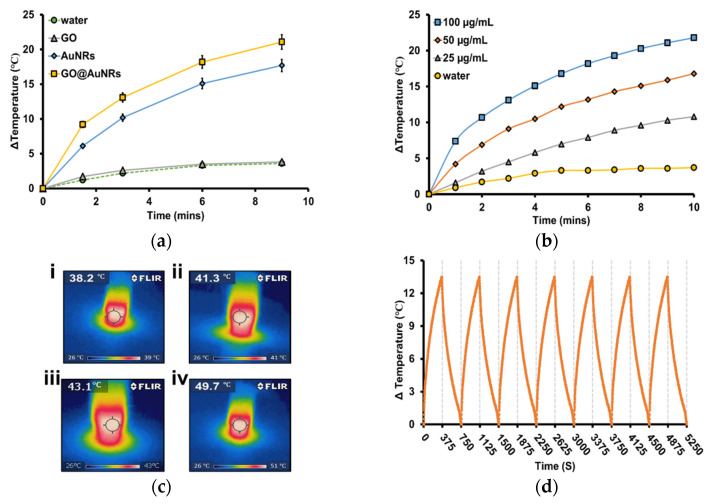
(**a**) Photothermal profile of water, GO, AuNRs and GO@AuNRs at 100 µg/mL irradiated with ~500 mW @ 785 nm Laser. (**b**) The photothermal efficiency of GO@AuNRs at different concentrations irradiated with ~500 mW @ 785 nm Laser. (**c**) IR-Thermal camera images of GO@AuNRs at different times (**i**) 2 min, (**ii**) 4 min, (**iii**) 6 min and (**iv**) 10 min at the concentration of 100 µg/mL. (**d**) Photothermal efficiency of GO@AuNRs at 50 µg/mL in an ON and OFF cycles with the intervals of 375s using ~500 mW @ 785 nm Laser.

GO, GO, GO@AuNRs and AuNRs at an equal concentration (100 µg/mL) were irradiated with 785 nm laser and ~500 mW to investigate the photothermal effect. The results showed an increase in the temperature with time (Figure 5a). The temperature change of GO@AuNRs increased by 21.1 °C within 9 min, whereas the temperature of the water, GO and AuNRs increased by 3.8, 3.8 and 17.7 °C, respectively. The higher increase in the temperature of GO@AuNRs when compared to bare AuNRs is due to the enhanced photon absorption when irradiated with a laser [20,43]. The water and GO showed almost similar temperature changes because GO has a highly oxidized structure with a disrupted π conjugation, which lowers its conductivity [6,20,43]. Figure 5b shows the temperature change of 10.8, 16.8 and 21.8 °C after 10 min of irradiation with the different concentrations of GO@AuNRs at 25, 50 and 100 µg/mL, respectively. The increase in temperature for the GO@AuNRs solution with time after irradiation was also observed on the IR-thermal camera, as shown in Figure 5c. The results showed a directly proportional relationship between irradiation time and increasing temperature. The GO@AuNRs were shown to be photothermal efficient when irradiated in ON and OFF laser cycles for 5250 s (Figure 5d). The GO@AuNRs were found to be stable during these cycles.

### 2.6. Effect of Culture Medium and pH on Optical Properties of GO@AuNRs-TMePyP

**Figure 6:**
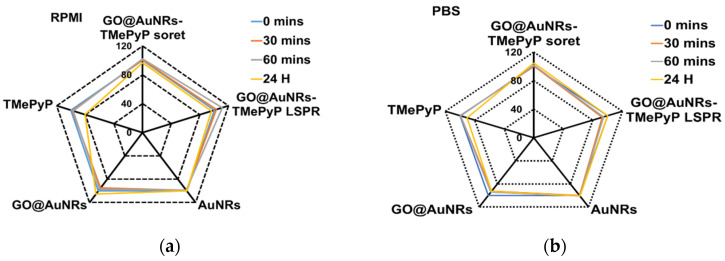

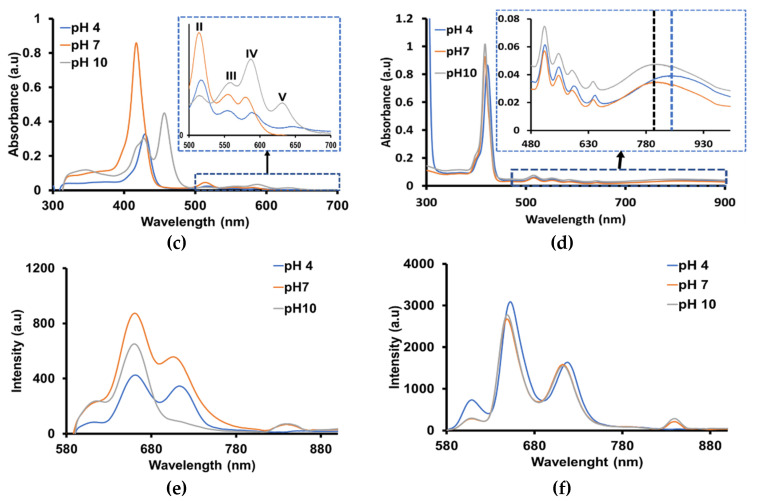
Time-dependent stability of AuNRs, GO@AuNRs, TMePyP and AuNRs-GO- TMePyP in (**a**) RPMI and (**b**) PBS. Stability of (**c**) TMePyP and (**d**) GO@AuNRs-TMePyP at different pH. The photoluminescent stability of (**e**) TMePyP and (**f**) GO@AuNRs-TMePyP at different pH.

The colloidal stability of nanomaterials is one the most important characterizations for their biological applications. This helps to understand the nanomaterial’s physicochemical properties when they interact with the complex biological media comprising of electrolytes proteins, lipids and many more [44]. Stability of AuNRs, GO@AuNRs, TMePyP and GO@AuNRs-TMePyP in the supplemented culture medium RPMI (Figure 6a) and PBS (Figure 6b) at a volume ratio of 1:10 (sample:medium) was evaluated against time. The AuNRs, GO@AuNRs and GO@AuNRs-TMePyP were found to be highly stable (above 90%) for 24 h in RPMI medium. On the other hand, the TMePyP sample showed stability slightly above 80% in the RPMI medium after 24 h. However, when the samples were dispersed in PBS, all of them showed stability above 90% after 24 h. GO@AuNRs-TMePyP nanocomposite was more stable than the bare TMePyP in PBS. The stability of GO@AuNRs-TMePyP nanocomposite is attributed to the modified GO as it has been reported that GO prevents the protein from absorbing in the physiological environments [45].

The effect of pH on the stability of TMePyP and GO@AuNRs-TMePyP was carried out at different buffer solutions with pH 4, 7 and 10. It is seen in the UV-Vis spectrum (Figure 6c) that the Soret band and Q bands (II, III and IV) were red-shifted to 430 nm, 517 nm, 592.5 and 649 nm, respectively, in the acidic medium, which could be due to protonation. In an alkaline medium, the TMePyP was deprotonated, the Soret band on the UV-Vis spectrum red-shifted, showing two soret peaks at 430 and 458 nm, while the Q-bands IV and V became more pronounced with a slight red-shifting to 590 nm and blue-shifting to 633 nm, respectively [46]. In addition, the Q band II absorbance decreased. The UV-Vis spectrum (Figure 6d) of GO@AuNRs-TMePyP maintained the soret and Q bands without significant changes in the spectra. In Figure 6e, the photoluminescent measurements of TMePyP showed similar patterns compared with its UV-Vis spectrum (Figure 6c), with weak emission peaks in both acidic and alkaline pHs. However, PL spectra of GO@AuNRs-TMePyP (Figure 6f) showed a similar pattern in all the pHs. These results confirmed the strong protection of TMePyP by GO@AuNRs, and hence it is not greatly affected by pH changes.

### 2.7. Fluorescence Quantum Yield and Singlet Oxygen Quantum Yield of GO@AuNRs-TMePyP

**Figure 7:**
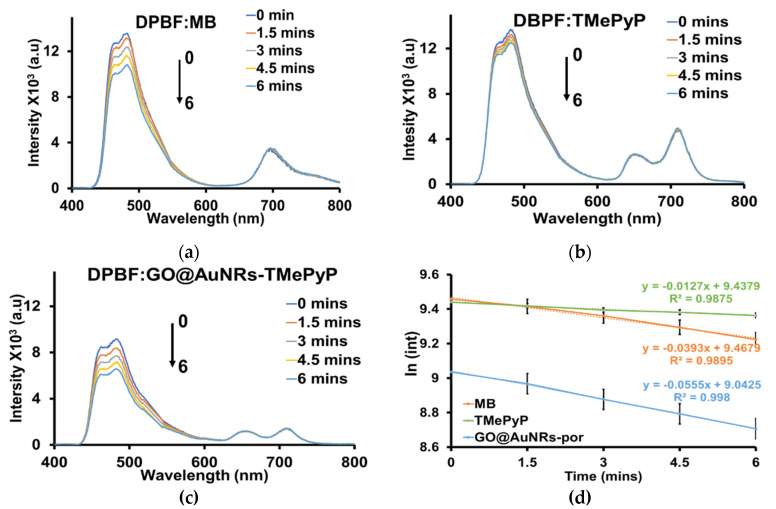
PL emission spectra of (**a**) DPBF:MB, (**b**) DPBF:TMePyP and (**c**) DPBF:GO@AuNRs-TMePyP excited at 560 nm for 6 min at the intervals of 1.5 min. (**d**) Integrated emission intensity time graph of DPBF at 471 nm after mixing with MB, TMePyP and GO@AuNRs-TMePyP and irradiated radiated at 560 nm for different time intervals 6.

The fluorescence quantum yield of TMePyP and GO@AuNRs-TMePyP were measured to be 0.31 and 0.14 using equation (2), respectively. The singlet oxygen emission quantum yield (SOQY) of TMePyP and GO@AuNRs-TMePyP were determined by a photochemical method using DPBF as singlet oxygen scavenger and MB as the reference. The singlet oxygen generation kinetics of mixed samples with DPBF were obtained by irradiating them at 535 nm at intervals of 1.5 min for 6 min and observing the intensity relative to the deterioration of DPBF at 471 nm (Figure 7). The singlet oxygen generation reaction rate constants were found to be 0.013 and 0.056 min^−1^ for TMePyP and GO@AuNRs-TMePyP, respectively, as calculated from first-order integrated rate reaction Equation (1) below:

(1) $$\ln{\left[\mathrm{int}\right]}_{t}=\;\ln{\left[\mathrm{int}\right]}_{0}-\mathrm{kt},$$

where [int]t is the intensity of DPBF (471 nm) at a particular irradiation time (t), while [int]0 is the initial intensity at the beginning of the reaction and k is the reaction rate constant, which is the slope of the graph.

The slopes of the graph in Figure 7d, together with Equation (3), were used to calculate the SOQY of TMePyP and GO@AuNRs-TMePyP. The SOQY of GO@AuNRs-TMePyP was more dramatically higher than that of the bare TMePyP. The SOQY of TMePyP increased from 0.094 to 0.186 after the formation of GO@AuNRs-TMePyP.

### 2.8. Cytotoxicity of GO@AuNRs-TMePyP

**Figure 8:**
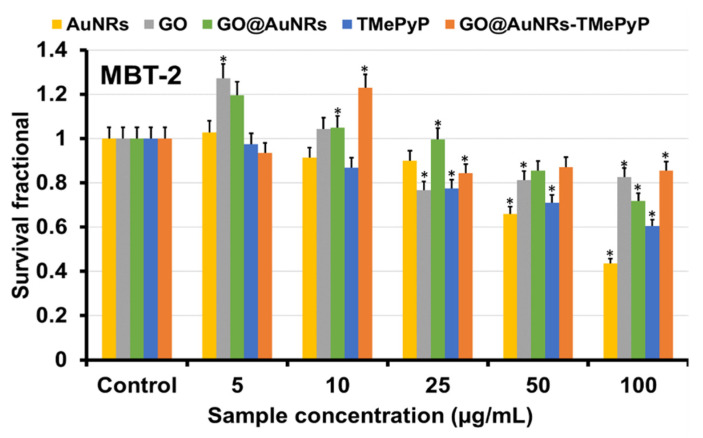
Cell viability of GO, AuNRs, GO@AuNRs, TMePyP and GO@AuNRs-TMePyP against MBT-2 cells (* *p* < 0.05).

The cytotoxicity of AuNRs, GO, GO@AuNRs, TMePyP and GO@AuNRs-TMePyP were evaluated against MBT-2 cell lines using the MTT assay. The GO shows cell viability above 80% in all concentrations used (5–100 µg/mL) (Figure 8). The AuNRs and TMePyP cell viability decreased with the increasing concentration (Figure 8). The decrease in the AuNRs cell viability has been attributed to the toxicity of the CTAB capping agent used during the synthesis [15,20,21,22,23]. However, GO modification of AuNRs improved cell viability by approximately 30% at 100 µg/mL (Figure 8). On the other hand, TMePyP showed 40% cell death at 100 µg/mL. However, GO@AuNRs-TMePyP showed an improved cell survival rate by 25% due to the suppression of TMePyP by GO@AuNRs. The cell survival rate results revealed that GO@AuNRs-TMePyP possesses great potential for biological applications because of its biocompatibility.

## 3. Materials and Methods

### 3.1. Materials

Hydrogen tetra-chloroauric hydrate (HAuCl_4_·H_2_O, 99.9%), sodium borohydride (NaBH_4_, 99%), silver nitrate (AgNO_3_, 99%), cetyltrimethylammonium bromide (CTAB, ≥99%), ascorbic acid (AA, 99%), sodium oleate, (NaOL, ≥99%), phosphate-buffered saline (PBS), Roswell Park Memorial Institute (RPMI) medium, hydrochloric acid (HCl, (12.1 M)), graphite powder, sulfuric acid (H_2_SO_4_), sodium nitrate (NaNO_3_), potassium permanganate (KMnO_4_), peroxide (H_2_O_2_), sodium hydroxide (NaOH), 3-pyridylcarboxaldehyde, propionic acid, pyrrole, sodium bicarbonate, ethanol, dichloromethane, methanol, ethyl acetate, n-hexane, chloroform, acetone, disodium hydrogen phosphate buffer, petroleum ether, calcium chloride, methylene blue, diethyl ether, a 1,3-diphenyl benzofuran and methyl iodide were purchased from Sigma-Aldrich, Kempton Park, South Africa. All solutions of gold salt, AgNO_3_ and NaBH_4_ were freshly prepared. All glassware used in the experiments were cleaned and washed thoroughly with MilliQ water (15.0 MΩ cm @ 25 °C) and dried before use.

### 3.2. Characterization Techniques

UV-Vis-NIR JASCO V-770 spectrophotometer (JASCO Corp., Japan) was used to measure UV-Vis-NIR absorption spectra of the samples. The surface chemistry was investigated using Spectrum two UATR spectrometer, Perkin Elmer (Beaconsfield, UK). The morphologies of the samples were captured by high-resolution transmission electron microscopy (HRTEM, JEOL 2010,200 KV, Tokyo, Japan). The size of the AuNRs and composites were measured from TEM images using ImageJ software. Zeta sizer (ELSZ-2000, Osaka, Japan) was used to measure the surface charge of the materials. Fluorescence spectra of the samples were measured using Shimadzu spectrophotometer (RF-6000, Kyoto, Japan). For NIR irradiation, XTRA II, High-Power, Single-Frequency Diode Laser (~500 mW @ 785 nm, Tokyo, Japan) with an 8 mm beam diameter was used.

### 3.3. Synthesis of 5,10,15,20-Tetrakis (3-Methyl Pyridyl) Porphyrin

5,10,15,20-tetrakis(3-pyridyl)porphyrin, TPyP was prepared using Adler-Longo method with modifications, which was further methylated to produce 5,10,15,20-tetrakis (3-methyl pyridyl) porphyrin (TMePyP) [47,48]. Initially, TPyP was synthesized by mixing 3.76 mL (0.04 mol) of 3-pyridylcarboxaldehyde and 2.8 mL (0.04 mol) of freshly distilled pyrrole in a flask containing 150 mL of propionic acid under reflux conditions. After ca. 30 min, the flask was cooled to room temperature, and distilled water (150 mL) was added to the reaction mixture. The product was extracted using 150 mL of dichloromethane. After removal of the solvent, the product was purified by column chromatography using dichloromethane, n-hexane, and methanol (1:1:1) as eluents to give 350 mg of TPyP yield.

For the methylation of TPyP, 330 mg of the as-synthesized TPyP was mixed with 30 mL of MeI in CHCl_3_ at room temperature and stirred. After 6 h, the solution was filtered and washed with diethyl ether, and the filtrate was air-dried. ^1^H NMR spectra and ^13^C-NMR spectra were recorded on a Bruker Advance DPX-400 MHz spectrometer (Karlsruhe, Germany). The absorption spectra were recorded on a UV-Vis-NIR JASCO V-770 spectrophotometer (JASCO Corp., Tokyo, Japan).

### 3.4. Synthesis of GO, AuNRs and Fabrication of GO@AuNRs

Graphene oxide was prepared using a modified Hummers’ method [39]. Briefly, 69 mL of ice-cold concentrated H_2_SO_4_ was added to a mixture of 3.01 g of graphite flakes and 1.51 g of NaNO_3_, while stirring in an ice bath. This was followed by the slow addition of KMnO_4_ (~10 g) and warmed to 35 °C while stirring for 30 min. Next, 138 mL of distilled water was added in portions, and the temperature was increased to 95 °C and stirred for 30 min. The solution was then cooled to room temperature, and the reaction was terminated by adding H_2_O_2_ (30 mL). The solution was left to settle, and the suspension was discarded, followed by pH neutralization with distilled water; it was then allowed to dry at room temperature. The obtained graphite oxide powder was exfoliated into GO sheets using ultrasonication.

AuNRs were synthesized using the seed-mediated method following the modified procedures developed by Vigderman and Zubarev [49]. Briefly, 0.250 mL of HAuCl_4_·H_2_O (0.010 M) and 9.5 mL of CTAB (0.10 M) was prepared. Next, 0.6 mL of freshly prepared ice-cold 0.010 M NaBH_4_ (prepared in NaOH (0.010 M)) was added, and the solution was stirred for 10 min. The solution was kept unstirred for an hour at room temperature. The growth solution was prepared by mixing 0.50 mL of HAuCl_4_·H_2_O (0.010 M), 8.0 mL of CTAB (0.10 M) and 0.04 mL of freshly prepared AgNO_3_ (0.10 M), respectively, with gentle stirring for 15 min. This was followed by the addition of HCl (1.0 M) to obtain a pH of 2.55. Next, 0.50 mL of 0.10 M hydroquinone was added, and the solutions turned completely colorless. Lastly, 2.0 mL of the seed solution was added, and the solution was left unstirred for 20 h at room temperature. After 20 h, the AuNRs were collected by centrifugation to remove excess CTAB and Au nanoparticles of other shapes (AuNPs) using different centrifugation speeds.

To fabricate GO@AuNRs nanocomposite, PVP (16 mg) was added to GO dispersion (4 mL, 0.1 mg/mL), followed by stirring for 30 min. Then, 2 mL of AuNRs dispersion was added to the PVP-stabilized GO solution under vigorous stirring for 24 h at room temperature. The resulting dispersion was washed and centrifuged three times to remove excess PVP and AuNRs. The final pellet was dissolved in 2 mL of deionized water.

### 3.5. Anchoring TMePyP on GO@AuNRs

The formation of GO@AuNRs nanocomposite-porphyrin conjugate was carried out using Tsolekile et al. [50] with modifications. Briefly, 0.2 mL of TMePyP (1 mg/10 mL, H_2_O) was added to 2 mL of GO@AuNRs and stirred for 1 h at room temperature.

### 3.6. Photothermal Evaluation

Photothermal efficiency of the AuNRs was measured by placing 1 mL of different concentrations (5–100 µg·mL^−1^) of AuNRs in a 1.5 mL Eppendorf tube followed by irradiation using an ~500 mW, 785 nm laser. For comparison, 100 µg·mL^−1^ of AuNRs, GO, GO@AuNRs and GO@AuNRs-TMePyP was irradiated using the same laser. For photothermal efficiency, reproducibility and photostability studies, 1 mL of AuNRs, GO@AuNRs and GO@AuNRs-TMePyP (all samples at 100 µg·mL^−1^) was irradiated in a 1.5 mL Eppendorf tube in an ON and OFF cycle with a time intervals of 6 min using an ~500 mW @ 785 nm laser. Photostability of all the samples after 1 h of irradiation was measured using UV-Vis-NIR spectroscopy. The temperature changes with time in all experiments were measured using a thermocouple.

### 3.7. Culture Medium and pH Stability Evaluation

TMePyP and GO@AuNRs-TMePyP were tested for stability at pH 4, 7 and 10 buffer solutions. Briefly, TMePyP and GO@AuNRs-TMePyP were added to the pH buffer solution at 1:10 volume ratio. The absorbance of each sample was measured using UV-Vis-NIR and PL spectroscopy. The stability of AuNRs, GO@AuNRs and AuNRs@GQDs was also tested in two culture media (RPMI and PBS, pH = 7.4). Briefly, 200 µL of AuNRs, GO@AuNRs and GO@AuNRs-TMePyP was added separately into 2 mL of RPMI (supplemented with 10% fetal bovine serum (FBS), 1% L-glutamine-penicillin-streptomycin (L-Glu), and 0.5% geneticin G418), and the absorbance at 850 nm and for TMePyP at 415 were measured immediately (30 min, 60 min and 24 h for all the samples). The same procedure was followed for the stability in phosphate-buffered solution (PBS, Ca^2+^ and Mg^2+^ free, pH = 7.4). The medium without samples was used as the reference samples.

### 3.8. Fluorescence Quantum Yield Measurements

TMePyP and GO@AuNRs-TMePyP were dispersed in distilled water to have an absorbance close to 0.1 at 560 nm, while methylene blue (MB) was dispersed in water to have an absorbance close to 0.1 at 560 nm. The samples were placed in a 3 mL pre-cleaned quartz cuvette with 1 cm optical path. The entire spectrum was scanned against the background spectrum of water. The emission spectra and quantum yield (QY) were obtained at ambient conditions using a Shimadzu RF-6000 (Shimadzu Corporation, Kyoto, Japan) spectrophotometer. The QY was calculated using the following Equation (2) below:

(2) $${\mathrm{QY}}_{s}=\left(\frac{\left(\frac{A_{s}}{A_{\mathrm{MB}}}\right)\times \left(\frac{{\mathrm{Abs}}_{\mathrm{MB}}}{{\mathrm{Abs}}_{s}}\right)}{\left(\frac{n_{\mathrm{MB}}^{2}}{n_{s}^{2}}\right)}\right)\times {\;\mathrm{QY}}_{\mathrm{MB}},$$

where QY_S_ and QY_MB_ are the quantum yield of the sample and reference, A_S_ and A_MB_ are the area under the high-intensity peaks of the sample and reference, Abs_S_ and Abs_MB_ are the absorbances at the excitation wavelength of the sample and reference, and n_S_ and n_MB_ are the refractive indexes of the sample and reference, respectively. The reference standard used was the MB solution in water with QY of 0.52.

### 3.9. Singlet Oxygen Quantum Yield Evaluation

Singlet oxygen quantum yield (SOQY) was determined following Tsolekile et al. [46]. In short, the standard of 1,3-diphenyl benzofuran (DPBF) solution (32 mM in DMSO), methylene blue (MB) solution (20 mM in DMSO), TMePyP solution (10 mM in water) and GO solution (0.1 mg/mL in water) were prepared, followed by a mixture of DPBF:TMePyP, DPBF:MB, DPBF:GO, DPBF:GO@AuNRs–TMePyP conjugate in a ratio of 1:1. The photoluminescence spectrophotometry laser light was used to irradiate the mixtures at 535 nm for 6 min with a 1.5 min time interval. The DPBF intensity decrease was monitored at 471 nm peak position. The SOQY was calculated against an aqueous solution of methylene blue (20 mM) as a standard (SOQY = 0.52) using Equation (3) below:

(3) $$\mathrm{SOQY}=\left(\frac{\left(\frac{I_{s}}{I_{\mathrm{MB}}}\right)\left(\frac{M_{s}}{M_{\mathrm{MB}}}\right)}{\left(\frac{{\mathrm{Abs}}_{s}}{{\mathrm{Abs}}_{\mathrm{MB}}}\right)}\right){\mathrm{SOQY}}_{\mathrm{MB}},$$

where SOQY and SOQY_MB_ are the SOQY of the samples (TMePyP, conjugate) and the reference (MB, 0.52), Mc and M_MB_ are the slopes of the samples and reference, Abs_s_ and Abs_MB_ are the absorbance of the samples and the reference at irradiation wavelength, and I_S_ and I_MB_ are the refractive indexes of the sample and reference, respectively.

### 3.10. Cell Culture, Cytotoxicity and Photo-Cytotoxicity Assays

The mouse bladder tumor line-2 (MBT-2) cell-line, donated by Tohoku University, was cultured in D-MEM (supplemented with 10% FBS). Cells were then incubated at 37 °C in a mixture of 5% carbon dioxide and 95% air until 80% confluence was achieved. Cytotoxicity assay was evaluated by following the standard 3-(4,5-Dimethylthiazol-2-yl)-2,5-Diphenyltetrazolium Bromide (MTT) assay protocol. Briefly, 2 mL of cell suspension (1.0 × 10^4^ cells·mL^−1^) was added to 12 wells plates separately and incubated for 24 h with the same condition as cell culture. After 24 h of incubation, cells were exposed to 100 µL of GO, AuNRs, GO@AuNRs, TMePyP and GO@AuNRs-TMePyP, at different concentrations (5–100 µg·mL^−1^) in triplicate, and further incubated for another 24 h. Control cells were not exposed to any samples. After 24 h, 0.2 mL of 3-(4,5-Dimethylthiazol-2-yl)-2,5-Diphenyltetrazolium Bromide (MTT (5 mg·mL^−1^)) solution was added and incubated at the same condition for 1 h. The medium was discarded in all wells, and 2 mL of dimethyl sulfoxide was added. Then, 0.1 mL from each well was transferred to a 96 well plate, and the absorbance was measured at a wavelength of 590 nm. The survival fraction for cytotoxicity assays was calculated from the following Equation (4):

(4) $$\mathrm{Survival}\;\mathrm{fraction}=\;\frac{A_{s\;}-{\;A}_{b}}{A_{c}\;-{\;A}_{b}},$$

where A_s_ is the absorbance of cells with the sample, A_b_ is the absorbance of the blank sample and A_c_ is the absorbance of the cell without sample as a control.

### 3.11. Statistical Analysis

Data are presented as the mean ± standard error of the mean (SEM). Statistical comparisons were made using Tukey’s multiple comparisons test. Statistically, values of *p* < 0.05 were significant.

## 4. Conclusions

In summary, a synergistic phototherapeutic agent was prepared with enhanced thermal and colloidal stability, and photothermal and photodynamic properties. TPyP was synthesized and methylated to TMePyP using Adler-Longo method and was characterized using different analytical techniques to confirm its properties. The GO was synthesized using a modified Hummers method and then further functionalized with PVP. The AuNRs were synthesized using the seed-mediated method and incorporated on modified GO’s surface to form GO@AuNRs. The optical and structural characterizations were used to confirm the properties of the GO, PVP-modified GO, AuNRs and GO@AuNRs. GO@AuNRs showed higher photothermal efficacy than bare AuNRs and GO, with excellent ON and OFF photothermal cycles. The TMePyP was then anchored on the surface of GO@AuNRs. GO@AuNRs-TMePyP exhibits good stability in both culture media (RPMI and PBS) and at different pH solutions, with more singlet oxygen production than bare TMePyP at a fast rate. The cytotoxicity of GO, AuNRs and TMePyP evaluated against MBT-2 cancer cell lines using MTT assay showed that GO@AuNRs-TMePyP was more biocompatible and less light-sensitive than bare TMePyP, even at a high concentration of 100 µg/mL. The results of this study showed GO@AuNRs-TMePyP has a potential as an agent for synergistic phototherapeutic action.

## Data Availability

Data is contained within the article and Supplementary Material.

## Supplementary Materials

The following are available online at https://www.mdpi.com/article/10.3390/ph14121295/s1, Scheme S1: Schematic diagram for the synthesis of TMePyP, Figure S1: ^1^H-NMR of TPyP, Figure S2: ^1^H-NMR of TMePyP. Figure S3: ^13^C-NMR of TPyP, Figure S4: ^13^C-NMR of TMePyP.

## References

[1] World Health Organization WHO Report on Cancer: Setting Priorities, Investing Wisely and Providing Care for All. https://apps.who.int/iris/handle/10665/330745.

[2] Zeng D., Wang L., Tian L., Zhao S., Zhang X., Li H. (2019). Synergistic photothermal/photodynamic suppression of prostatic carcinoma by targeted biodegradable MnO2 nanosheets. Drug Deliv..

[3] Xie Z., Fan T., An J., Choi W., Duo Y., Ge Y., Zhang B., Nie G., Xie N., Zheng T. (2020). Emerging combination strategies with phototherapy in cancer nanomedicine. Chem. Soc. Rev..

[4] Qiao J., Tian F., Deng Y., Shang Y., Chen S., Chang E., Yao J. (2020). Bio-orthogonal click-targeting nanocomposites for chemo-photothermal synergistic therapy in breast cancer. Theranostics.

[5] Wu R., Wang H., Hai L., Wang T., Hou M., He D., He X., Wang K. (2020). A photosensitizer-loaded zinc oxide-polydopamine core-shell nanotherapeutic agent for photodynamic and photothermal synergistic therapy of cancer cells. Chin. Chem. Lett..

[6] Lebepe T.C., Parani S., Oluwafemi O.S. (2020). Graphene Oxide-Coated Gold Nanorods: Synthesis and Applications. Nanomaterials.

[7] Banstola A., Pham T.T., Jeong J.-H., Yook S. (2019). Polydopamine-tailored paclitaxel-loaded polymeric microspheres with adhered NIR-controllable gold nanoparticles for chemo-phototherapy of pancreatic cancer. Drug Deliv..

[8] Dembereldorj U., Choi S.Y., Ganbold E.O., Song N.W., Kim D., Choo J., Lee S.Y., Kim S., Joo S.W. (2014). Gold Nanorod-Assembled PEGylated Graphene-Oxide Nanocomposites for Photothermal Cancer Therapy. Photochem. Photobiol..

[9] Lau I.P., Chen H., Wang J., Ong H.C., Leung K.C.-F., Ho H.P., Kong S.K. (2012). In vitro effect of CTAB-and PEG-coated gold nanorods on the induction of eryptosis/erythroptosis in human erythrocytes. Nanotoxicology.

[10] Liopo A.V., Conjusteau A., Oraevsky A.A. (2012). PEG-coated gold nanorod monoclonal antibody conjugates in preclinical research with optoacoustic tomography, photothermal therapy, and sensing. Photons Plus Ultrasound: Imaging and Sensing 2012.

[11] Ma X., Cheng Y., Huang Y., Tian Y., Wang S., Chen Y. (2015). PEGylated gold nanoprisms for photothermal therapy at low laser power density. RSC Adv..

[12] Niidome T., Yamagata M., Okamoto Y., Akiyama Y., Takahashi H., Kawano T., Katayama Y., Niidome Y. (2006). PEG-modified gold nanorods with a stealth character for in vivo applications. J. Control. Release.

[13] Schulz F., Friedrich W., Hoppe K., Vossmeyer T., Weller H., Lange H. (2016). Effective PEGylation of gold nanorods. Nanoscale.

[14] Yang H., He H., Tong Z., Xia H., Mao Z., Gao C. (2020). The impact of size and surface ligand of gold nanorods on liver cancer accumulation and photothermal therapy in the second near-infrared window. J. Colloid Interface Sci..

[15] Qi Z., Shi J., Zhu B., Li J., Cao S. (2020). Gold nanorods/graphene oxide nanosheets immobilized by polydopamine for efficient remotely triggered drug delivery. J. Mater. Sci..

[16] Oladipo A.O., Lebepe T.C., Ncapayi V., Tsolekile N., Parani S., Songca S.P., Mori S., Kodama T., Oluwafemi O.S. (2021). The Therapeutic Effect of Second Near-Infrared Absorbing Gold Nanorods on Metastatic Lymph Nodes via Lymphatic Delivery System. Pharmaceutics.

[17] Borri C., Centi S., Ratto F., Pini R. (2018). Polylysine as a functional biopolymer to couple gold nanorods to tumor-tropic cells. J. Nanobiotechnol..

[18] Gonçalves P.J., Bezerra F.C., Almeida L.M., Alonso L., Souza G.R.L., Alonso A., Zílio S.C., Borissevitch I.E. (2019). Effects of bovine serum albumin (BSA) on the excited-state properties of meso-tetrakis(sulfonatophenyl) porphyrin (TPPS4). Eur. Biophys. J..

[19] Liu K., Zheng Y., Lu X., Thai T., Lee N.A., Bach U., Gooding J.J. (2015). Biocompatible gold nanorods: One-step surface functionalization, highly colloidal stability, and low cytotoxicity. Langmuir.

[20] Robinson J.T., Tabakman S.M., Liang Y., Wang H., Sanchez Casalongue H., Vinh D., Dai H. (2011). Ultrasmall Reduced Graphene Oxide with High Near-Infrared Absorbance for Photothermal Therapy. J. Am. Chem. Soc..

[21] Sun B., Wu J., Cui S., Zhu H., An W., Fu Q., Shao C., Yao A., Chen B., Shi D. (2017). In situ synthesis of graphene oxide/gold nanorods theranostic hybrids for efficient tumor computed tomography imaging and photothermal therapy. Nano Res..

[22] Turcheniuk K., Dumych T., Bilyy R., Turcheniuk V., Bouckaert J., Vovk V., Chopyak V., Zaitsev V., Mariot P., Prevarskaya N. (2016). Plasmonic photothermal cancer therapy with gold nanorods/reduced graphene oxide core/shell nanocomposites. RSC Adv..

[23] Wei Q., Ni H., Jin X., Yuan J. (2015). Graphene oxide wrapped gold nanorods for enhanced photo-thermal stability. RSC Adv..

[24] Wang J., Zhu G., You M., Song E., Shukoor M.I., Zhang K., Altman M.B., Chen Y., Zhu Z., Huang C.Z. (2012). Assembly of Aptamer Switch Probes and Photosensitizer on Gold Nanorods for Targeted Photothermal and Photodynamic Cancer Therapy. ACS Nano.

[25] Wang N., Zhao Z., Lv Y., Fan H., Bai H., Meng H., Long Y., Fu T., Zhang X., Tan W. (2014). Gold nanorod-photosensitizer conjugate with extracellular pH-driven tumor targeting ability for photothermal/photodynamic therapy. Nano Res..

[26] Tham H.P., Chen H., Tan Y.H., Qu Q., Sreejith S., Zhao L., Venkatraman S.S., Zhao Y. (2016). Photosensitizer anchored gold nanorods for targeted combinational photothermal and photodynamic therapy. Chem. Commun..

[27] Wang B., Wang J.-H., Liu Q., Huang H., Chen M., Li K., Li C., Yu X.-F., Chu P.K. (2014). Rose-bengal-conjugated gold nanorods for in vivo photodynamic and photothermal oral cancer therapies. Biomaterials.

[28] Luo T., Qian X., Lu Z., Shi Y., Yao Z., Chai X., Ren Q. (2015). Indocyanine green derivative covalently conjugated with gold nanorods for multimodal phototherapy of fibrosarcoma cells. J. Biomed. Nanotechnol..

[29] Ferreira D.C., Monteiro C.S., Chaves C.R., Sáfar G.A.M., Moreira R.L., Pinheiro M.V.B., Martins D.C.S., Ladeira L.O., Krambrock K. (2017). Hybrid systems based on gold nanostructures and porphyrins as promising photosensitizers for photodynamic therapy. Colloids Surf. B Biointerfaces.

[30] Choi J., Kim S.Y. (2020). Photothermally enhanced photodynamic therapy based on glutathione-responsive pheophorbide a-conjugated gold nanorod formulations for cancer theranostic applications. J. Ind. Eng. Chem..

[31] Wang J., Tang H.Y., Yang W.L., Chen J.Y. (2012). Aluminum phthalocyanine and gold nanorod conjugates: The combination of photodynamic therapy and photothermal therapy to kill cancer cells. J. Porphyr. Phthalocyanines.

[32] Huang Q., Chen Y., Hao L., Zhou R., Li Y., Li Q., Zhu B., Cai X. (2020). Pegylated carbon nitride nanosheets for enhanced reactive oxygen species generation and photodynamic therapy under hypoxic conditions. Nanomed. Nanotechnol. Biol. Med..

[33] Zheng X.-H., Nie X., Liu H.-Y., Fang Y.-M., Zhao Y., Xia L.-X. (2016). TMPyP4 promotes cancer cell migration at low doses, but induces cell death at high doses. Sci. Rep..

[34] Kruk N. (2006). Fluorescent properties and symmetry of the monodeprotonated form of 5, 10, 15, 20-tetrakis-(4-N-methylpyridyl)-porphyrin. J. Appl. Spectrosc..

[35] Zagami R., Franco D., Pipkin J.D., Antle V., De Plano L., Patanè S., Guglielmino S., Monsù Scolaro L., Mazzaglia A. (2020). Sulfobutylether-β-cyclodextrin/5,10,15,20-tetrakis(1-methylpyridinium-4-yl)porphine nanoassemblies with sustained antimicrobial phototherapeutic action. Int. J. Pharm..

[36] Zakavi S., Rahiminezhad H., Ghanbelanie Mojarrad A., Mokary Yazdeli T., Alizadeh R. (2013). Effects of Core and/or Peripheral Protonation of *meso*-Tetra(2-, 3-, and 4-pyridyl)Porphyrin and *meso*-Tetra(3-methylpyridyl)Porphyrin on Their UV-vis Spectra. J. Spectrosc..

[37] Wu X., Field R., Wu J.J., Zhang K. (2017). Polyvinylpyrrolidone modified graphene oxide as a modifier for thin film composite forward osmosis membranes. J. Membr. Sci..

[38] Huang P., Wang S., Wang X., Shen G., Lin J., Wang Z., Guo S., Cui D., Yang M., Chen X. (2015). Surface Functionalization of Chemically Reduced Graphene Oxide for Targeted Photodynamic Therapy. J. Biomed. Nanotechnol..

[39] Lebepe T.C., Parani S., Vuyelwa N., Kodama T., Oluwafemi O.S. (2020). Cytotoxicity evaluation of Graphene Oxide against Adherent and Suspension cancer cells. Mater. Lett..

[40] Su G., Yang C., Zhu J.-J. (2015). Fabrication of gold nanorods with tunable longitudinal surface plasmon resonance peaks by reductive dopamine. Langmuir.

[41] Umeyama T., Mihara J., Tezuka N., Matano Y., Stranius K., Chukharev V., Tkachenko N.V., Lemmetyinen H., Noda K., Matsushige K. (2012). Preparation and photophysical and photoelectrochemical properties of a covalently fixed porphyrin–chemically converted graphene composite. Chem. A Eur. J..

[42] Xu Y., Liu Z., Zhang X., Wang Y., Tian J., Huang Y., Ma Y., Zhang X., Chen Y. (2009). A graphene hybrid material covalently functionalized with porphyrin: Synthesis and optical limiting property. Adv. Mater..

[43] Khan M.S., Pandey S., Bhaisare M.L., Gedda G., Talib A., Wu H.-F. (2017). Graphene oxide@gold nanorods for chemo-photothermal treatment and controlled release of doxorubicin in mice Tumor. Colloids Surf. B Biointerfaces.

[44] Moore T.L., Rodriguez-Lorenzo L., Hirsch V., Balog S., Urban D., Jud C., Rothen-Rutishauser B., Lattuada M., Petri-Fink A. (2015). Nanoparticle colloidal stability in cell culture media and impact on cellular interactions. Chem. Soc. Rev..

[45] Sheng Y., Tang X., Peng E., Xue J. (2013). Graphene oxide based fluorescent nanocomposites for cellular imaging. J. Mater. Chem. B.

[46] Tsolekile N., Ncapayi V., Obiyenwa G.K., Matoetoe M., Songca S., Oluwafemi O.S. (2019). Synthesis of meso-tetra-(4-sulfonatophenyl) porphyrin (TPPS4)–CuInS/ZnS quantum dots conjugate as an improved photosensitizer. Int. J. Nanomed..

[47] Kitaoka S., Nobuoka K., Ihara K., Ishikawa Y. (2014). A simple method for efficient synthesis of tetrapyridyl-porphyrin using Adler method in acidic ionic liquids. RSC Adv..

[48] Peng C.-L., Lai P.-S., Chang C.-C., Lou P.-J., Shieh M.-J. (2010). The synthesis and photodynamic properties of meso-substituted, cationic porphyrin derivatives in HeLa cells. Dyes Pigments.

[49] Vigderman L., Zubarev E.R. (2013). High-yield synthesis of gold nanorods with longitudinal SPR peak greater than 1200 nm using hydroquinone as a reducing agent. Chem. Mater..

[50] Tsolekile N., Nahle S., Zikalala N., Parani S., Sakho E.H.M., Joubert O., Matoetoe M.C., Songca S.P., Oluwafemi O.S. (2020). Cytotoxicity, fluorescence tagging and gene-expression study of CuInS/ZnS QDS—meso (hydroxyphenyl) porphyrin conjugate against human monocytic leukemia cells. Sci. Rep..

