# Supplementary material for: Graphene Oxide-Gold Nanorods Nanocomposite-Porphyrin Conjugate as Promising Tool for Cancer Phototherapy Performance
Source: Pharmaceuticals (Basel). 2021 Dec 11;14(12):1295. doi: 10.3390/ph14121295 (PMC8706362; doi:10.3390/ph14121295)
Supplement: Supplementary file 1 [file pharmaceuticals-14-01295-s001.zip › pharmaceuticals-1473810-supplementary.pdf]

Graphene Oxide-Gold Nanorods Nanocomposite-Porphyrin Conjugate as Promising Tool for Cancer Phototherapy Performance

Thabang Calvin Lebepe [1,2], Sundararajan Parani [1,2], Vuyelwa Ncapayi [1,2], Rodney Maluleke [1,2], Grace It Mwad Mbaz [1,2], Olufunto Tolulope Fanoro [2], Jose Rajendran Varghese [1,2], Atsuki Komiya [3], Tetsuya Kodama [4] and Oluwatobi Samuel Oluwafemi [1,2,*]

1 Department of Chemical Science, University of Johannesburg, Johannesburg 2028, South Africa; calvyn.tl@gmail.com (T.C.L.), sbarani416@gmail.com (S.P.); vuyelwa.tito.ncapayi530@gmail.com (V.N.); rodney.maluleke@gmail.com (R.M.); gmbazitmwad@gmail.com (G.I.M.M.); josv3209@gmail.com (J.R.V.)
2 Centre for Nanomaterials Sciences Research, University of Johannesburg, Johannesburg 2028, South Africa; jolufunto@gmail.com
3 Graduate School of Engineering, Institute of Fluid Science, Tohoku University, Sendai 980-8577, Japan; komiya@tohoku.ac.jp
4 Graduate School of Biomedical Engineering, Tohoku University, Sendai 980-8575, Japan; kodama@tohoku.ac.jp
* Correspondence: oluwafemi.oluwatobi@gmail.com

SUPPORTING DOCUMENTS

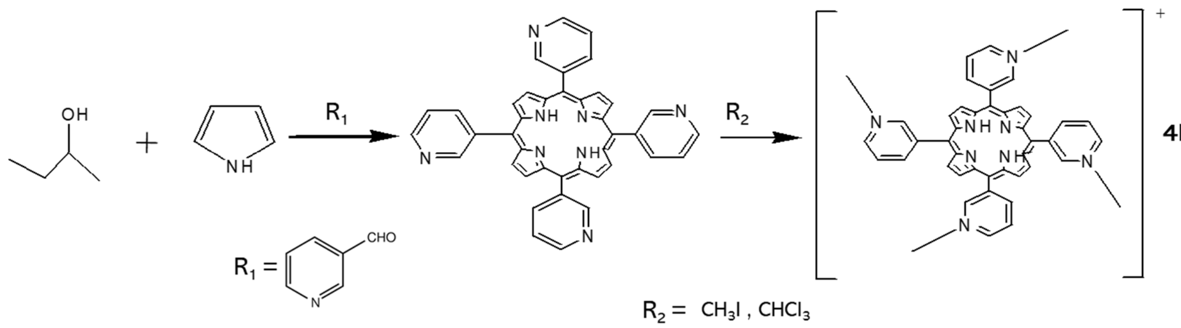

-

Scheme S1. Schematic diagram for the synthesis of TMePyP.

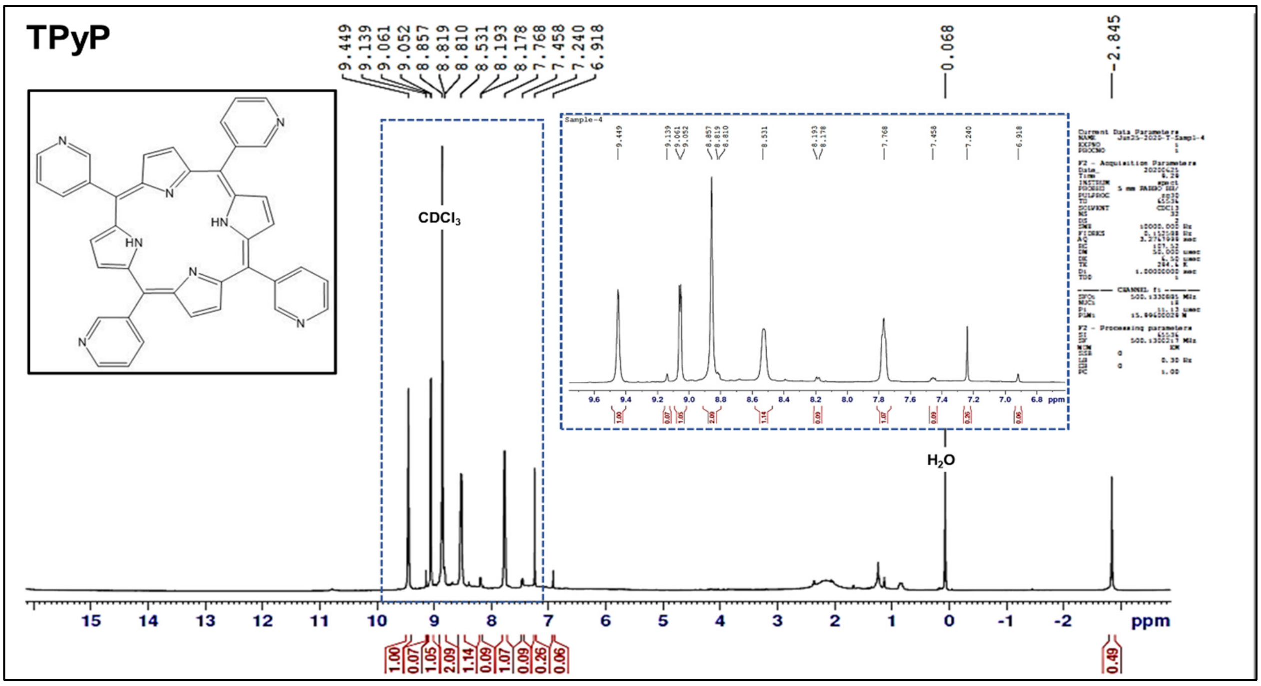

Figure S1. ^{1}H-NMR of TPyP.

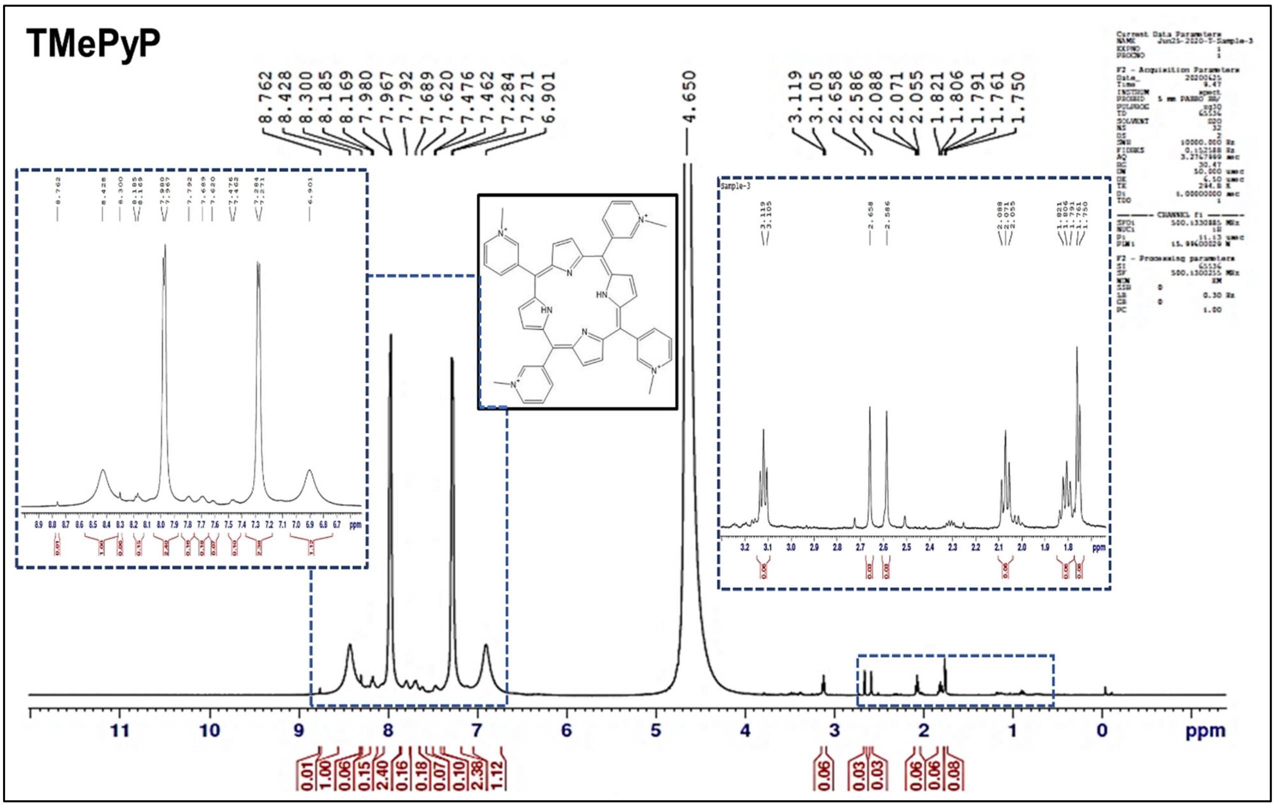

Figure S2. ^{1}H-NMR of TMePyP.

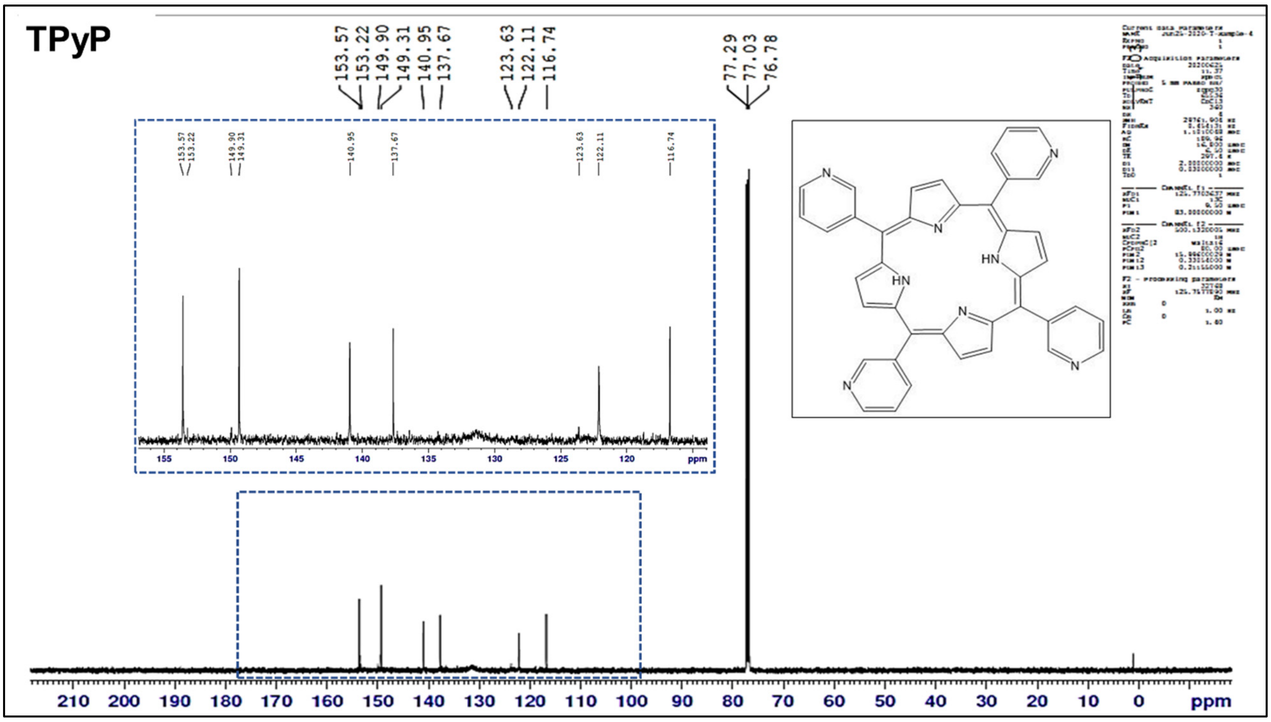

Figure S3. ^{13}C-NMR of TPyP.

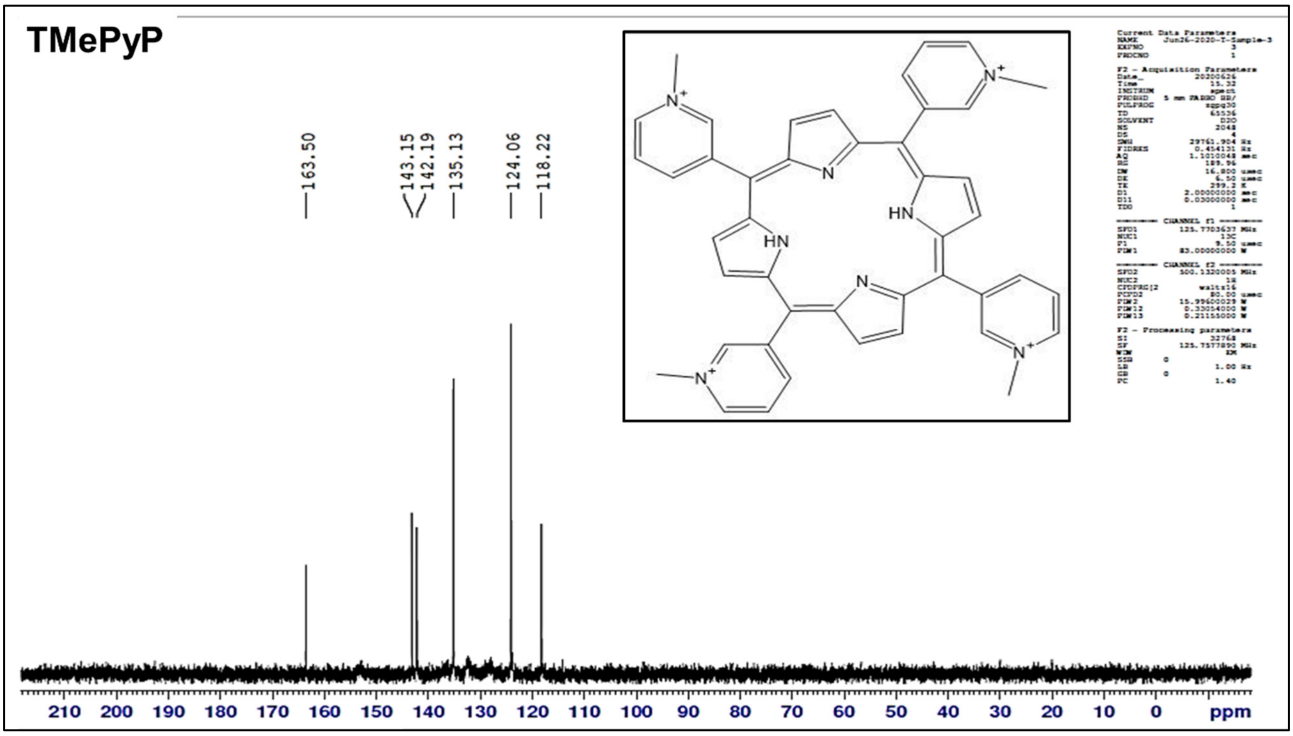

Figure S4. ^{13}C-NMR of TMePyP.
